# Genome-Wide Identification and Expression Analysis of LBD Transcription Factor Genes in Passion Fruit (*Passiflora edulis*)

**DOI:** 10.3390/ijms23094700

**Published:** 2022-04-24

**Authors:** Jianxiang Liang, Zhimin Hou, Jingyi Liao, Yuan Qin, Lulu Wang, Xiaomei Wang, Weiqiang Su, Zhaoyan Cai, Yunying Fang, Mohammad Aslam, Yan Cheng, Ping Zheng

**Affiliations:** 1 Basic Forestry and Proteomics Center (BFPC), Fujian Provincial Key Laboratory of Haixia Applied Plant Systems Biology, College of Forestry, Fujian Agriculture and Forestry University, Fuzhou 350002, China; jxliang_china@126.com; 2 Center for Genomics and Biotechnology, Fujian Provincial Key Laboratory of Haixia Applied Plant Systems Biology, College of Life Science, Fujian Agriculture and Forestry University, Fuzhou 350002, China; Summer20200505@163.com (Z.H.); 13860028580@163.com (J.L.); yuanqin@fafu.edu.cn (Y.Q.); luluwanghn@163.com (L.W.); aslampmb1@gmail.com (M.A.); chengyan1220@hotmail.com (Y.C.); 3 State Key Laboratory of Ecological Pest Control for Fujian and Taiwan Crops, College of Plant Protection, Fujian Agriculture and Forestry University, Fuzhou 350002, China; 4 Guangxi Key Laboratory of Sugarcane Biology, College of Agriculture, Guangxi University, Nanning 530004, China; 5 Horticulture Research Institute, Guangxi Academy of Agricultural Sciences, Nanning Investigation Station of South Subtropical Fruit Trees, Ministry of Agriculture, Nanning 530004, China; wangxiaomei159@163.com (X.W.); nkyswq@sina.com (W.S.); caizhaoyan424@163.com (Z.C.); 6 College of Agriculture, Fujian Agriculture and Forestry University, Fuzhou 350002, China; 3200537016@fafu.edu.cn

**Keywords:** passion fruit, *LBD* transcription factor, floral development, target genes, gene expression

## Abstract

The lateral organ boundary domain (*LBD*) gene is a plant-specific transcription factor that plays a crucial role in plant growth and development, including the development of lateral vegetative organs such as leaf and root development, as well as floral organs such as sepal, petal, and pollen development. Passion fruit is a tropical fruit with important agricultural, economic and ornamental value. However, there is no systematic research report available on the *LBD* gene family of passion fruit. In this study, a genome-wide analysis of passion fruit *LBD* genes identified 33 *PeLBDs* that were unevenly distributed across nine chromosomes. According to phylogenetic and gene structure analysis, *PeLBDs* were divided into two categories: Class I (27) and Class II (6). Homologous protein modeling results showed that the gene members of the two subfamilies were structurally and functionally similar. *Cis*-acting element and target gene prediction analysis suggested that *PeLBDs* might participate in various biological processes by regulating diverse target genes involved in growth and development, metabolism, hormones and stress response. Collinearity analysis indicated that the expansion of the *PeLBD* gene family likely took place mainly by segmental duplication, and some duplicated gene pairs such as *PeLBD13/15* might show functional redundancy, while most duplicated gene pairs such as *PeLBD8/12* showed different expression profiles indicating their functional diversification. After filtering low expressed genes, all Class Id *PeLBD*s were more highly expressed during pollen development. At the same, all Class Ic and many other *PeLBDs* were relatively highly expressed during ovule development, similar with their homologous *LBD* genes in Arabidopsis, indicating their potential regulatory roles in reproductive tissue development in passion fruit. *PeLBDs* that were highly expressed in floral tissues were also expressed at a higher level in tendrils with some differences, indicating the close relationships of tendrils to floral tissues. Some genes such as *PeLBD23/25* might be simultaneously related to floral development and leaf early formation in passion fruit, while other *PeLBDs* showed a strong tissue-specific expression. For example, *PeLBD17/27/29* were specifically expressed in floral tissues, while *PeLBD11* were only highly expressed in fruit, suggesting their specific function in the development of certain tissues. A qRT-PCR was conducted to verify the expression levels of six *PeLBDs* in different tissues. Our analysis provides a basis for the functional analysis of *LBD* genes and new insights into their regulatory roles in floral and vegetative tissue development.

## 1. Introduction

Passion fruit (*Passiflora edulis*) is a perennial evergreen climbing vine that is widely cultivated in tropical and subtropical areas of the world due to its important edible, medicinal and ornamental value [1]. Its egg-shaped fruit is characterized by yellow juice, acid pulp, rich aroma and distinctive flavor, which is popular with numerous consumers. Due to the passion fruit being rich in flavonoids, alkaloids and other biologically active ingredients [2], the extracts of leaves, fruits, peels and seeds have certain medicinal values such as calming, antioxidant, anti-inflammatory, anxiolytic and anticancer [3,4,5]. As to its ornamental value, many varieties of passion fruit are used as ornamental plants and for flower racks as most of them have large floral organs, bright coronal filaments, a rich fragrance and luxuriant branches and leaves [6]. Taken together, passion fruit is an important crop, and the identification of related gene families are of great significance to the development of the global agricultural economy.

The lateral organ boundary domains (*LBD*) gene family, also known as the *ASYMMETRIC LEAVES2-like (AS2)* gene family, is a class of plant-specific transcription factor (TFs) found only in higher plants with key roles in the regulation of plant organ development [7,8]. The first *LBD* gene was identified in a screen for gene-trap expression patterns in the shoot apex of Arabidopsis seedlings. Recent investigations showed that LBD proteins are involved in many aspects of plant organ or tissue growth and development, including lateral roots, stems, leaves, embryo sacs, inflorescences and flowers [9,10,11], as well as physiological and biochemical processes such as penicillin biosynthesis, nitrogen metabolism and lateral organ development [12,13,14,15]. In Arabidopsis, the heterodimeric interactions between *ASYMMETRIC LEAVES1* and *2* (*AS1* and *AS2*) and *JAGGED LATERAL ORGANS* (*JLO*) proteins are implicated in the establishment of organ boundaries [16]. The AS1(*AtLBD36*), AS2 (*AtLBD6*) and *JAGGED* (*JAG*) genes negatively regulate boundary-specifying genes to promote sepal and petal development [10]; *AtLBD27/SIDECAR POLLEN (SCP)* plays a key role in the asymmetric division of microspores, and a combinatorial role of *AtLBD10* with *AtLBD27* is crucial for male gametophyte development in Arabidopsis [17]. *AtLBD16*, *AtLBD18* and *AtLBD29* together participate in lateral roots’ development in which *AtLBD29* is also involved in the auxin signaling process that regulates fiber wall biosynthesis [18]. *AtLBD16*, *AtLBD17*, *AtLBD18* and *AtLBD29* are also key regulators involved in the callus induction process related to plant regeneration [19].

*LBD* transcription factors contain three specific conserved domains arranged from N to C terminus: the zinc finger-like C-block (CX2CX6CX3C), the Gly-Ala-Ser-block (GAS-block) and the leucine-like zipper module (LX6LX3LX6L) [20]. Among them, the C-block contains four highly conserved cysteine motifs, which are essential elements for DNA binding. The GAS-block is located in the middle of the LOB structural domain and its conserved proline residues play key roles in the biological function of LBD proteins in Arabidopsis [15]. According to the structural characteristics of the *LBD* gene family, it can be divided into two subfamilies (Class I and Class II) [20,21]. Class I LBD proteins contain the CX2CX6CX3C zinc finger-like motif, the GAS-block and the leucine zipper module, which can be grouped into four clades (Ia, Ib, Ic and Ie). By contrast, Class II LBD proteins contain only the conserved zinc finger-like structural domain which can be grouped into two clades (IIa and IIb) [22]. Functional analysis in Arabidopsis, rice and other plants shows that Class I *LBD* genes are mainly involved in plant development, such as lateral root, leaf and flower development [11,20]. In contrast, Class II *LBD* genes might be involved in metabolic processes such as anthocyanin synthesis and additional nitrogen responses [11,13,23].

Due to the rapid development of genome sequencing technology, the *LBD* gene family has been comprehensively and systematically studied at the genome-wide level in some models or plants of agroforestry importance, such as Arabidopsis (*Arabidopsis thaliana*) [18], rice (*Oryza sativa* L.) [24], tomato (*Solanum lycopersicum* Mill.) [25], grape (*Vitis vinifera* L.) [26], pepper (*Capsicum annuum* L.) [27] and poplar (*Populus trichocarpa* Torr. and Gray) [28]. Passion fruit are characterized by large floral organs, bright coronal filaments, special tendrils and developed root systems, but the characteristics and roles of the *LBD* genes of passion fruit remains unclear. The release of the passion fruit genome provides a research basis for the identification of the *LBD* gene family at the genome-wide level. Therefore, in this study, we performed genome-wide identification, evolutionary relationship, chromosomal localization, collinearity analysis and comprehensive analysis of expression patterns of the passion fruit *LBD* gene family by bioinformatics methods to provide a theoretical basis for future research on the functional characterization of the passion fruit *LBD* gene family.

## 2. Results

### 2.1. Whole-Genome Characterization of PeLBD Genes in Passion Fruit

In this study, we first used the plant LBD-type LOB model (PF03195) to perform a hidden Markov model search on the whole-genome protein sequence of passion fruit. Secondly, to further verify the reliability of the screened candidate *LBD* gene family members, we used SMART and NCBI-CDD and detected the integrity of the LOB domain of the candidate protein. Finally, we removed redundant irrelevant genes and obtained 33 *LBD* gene family members (Table 1), which were renamed *PeLBD1*—*PeLBD31*, according to their positional order on the chromosomes. Two *LBD* genes (P_eduliaContig20022702.g and P_eduliaContig60022431.g) that could not be mapped to any chromosomal scaffold were renamed *PeLBD32* and *PeLBD33*, respectively. The 33 *PeLBD* genes were divided into two groups according to the presence/absence of the LX6LX3LX6L leucine zipper-like domain of the LBD protein: 27 members belonged to Class I and six members belonged to Class II. In addition, we further analyzed the physicochemical properties of these 33 LBD proteins. The proteins encoded by the 33 *LBD* genes contained 95 (*PeLBD7*) to 337 (*PeLBD17*) amino acids with molecular weights (MWs) ranging from 10,772.56 Da (*PeLBD7*) to 38,858.07 Da (*PeLBD17*). Predicted protein isoelectric points (pI) ranged from 4.5 (*PeLBD22*) to 9.64 (*PeLBD7*). Instability index calculations predicted that all LBD proteins were unstable in vitro. The aliphatic amino acid index (A.I.) showed that the protein thermal stability was between 57.58 (*PeLBD7*) and 92.37 (*PeLBD1*), indicating that their thermal stability differences were small. The grand average of hydropathicity score (GRAVY) for all LBD proteins was negative, indicating that they are predominantly hydrophilic. Finally, subcellular localization prediction showed that all LBD proteins were localized in the nucleus (Table S1).

### 2.2. Multiple Sequence Alignment and Phylogenetic Analysis of PeLBD Genes

**Figure 1:**
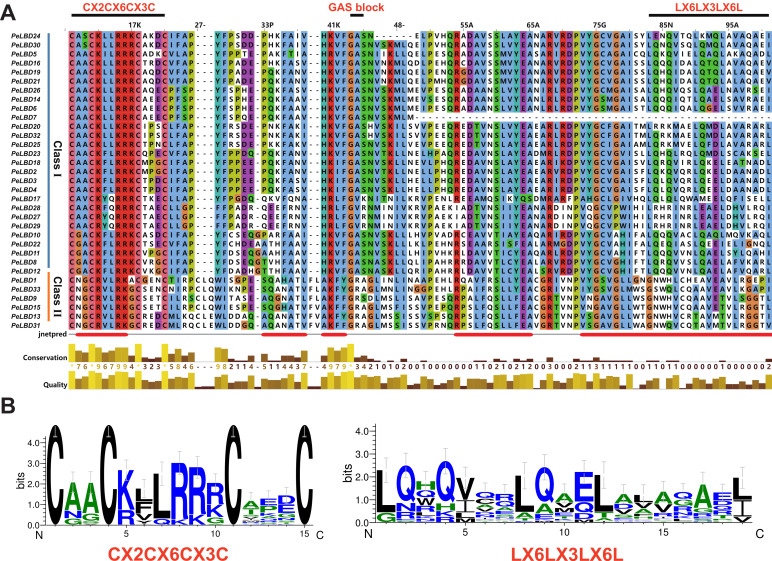
Multiple sequence alignment of the conserved domain proteins of the passion fruit *LBD* gene family. (**A**) The CX2CX6CX3C zinc finger-like domain was present in all 33 predicted PeLBD protein sequences, whereas the leucine zipper-like motif (LX6LX3LX6L) was present only in the Class I PeLBD proteins; (**B**) Conserved motif logos generated by the WebLogo program.

The conserved domains and phylogenetic relationships of 33 PeLBD proteins were explored by multiple sequence alignment of their LOB domains (CX2CX6CX3C). According to the results (Table S2), all LBD family members contain a highly conserved LOB region at the N-terminus, consisting of about 100 amino acids, and a zinc finger domain (Figure 1). The leucine zipper-like domain (LX6LX3LX6L) is only present in Class I PeLBD proteins, similar to the results of other plants studied. The predicted protein secondary structure contains only five *α*-helix bundles.

**Figure 2:**
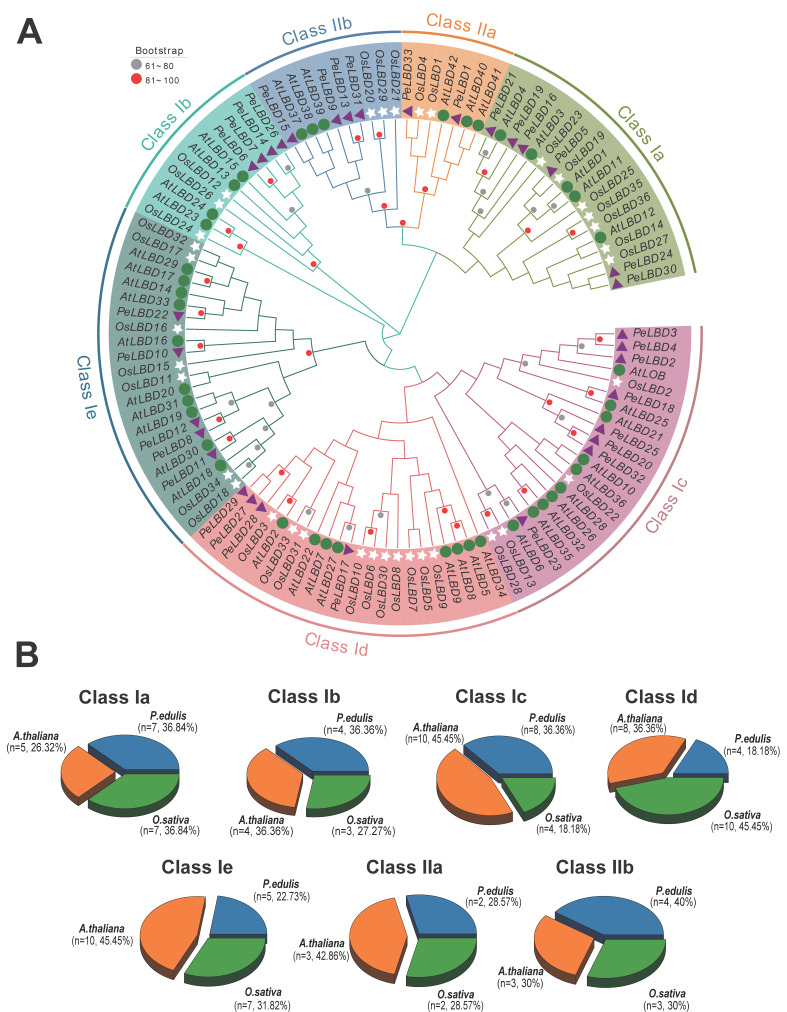
Phylogenetic tree of LBD proteins from passion fruit (Pe, *Passiflora edulis*), Arabidopsis (At, *Arabidopsis thaliana*) and rice (Os, *Oryza sativa*). (**A**) A phylogenetic tree of the LBD protein family was constructed by MEGA 11 software using the maximum likelihood (ML) option with 1000 bootstrap replicates. Purple triangles, green circles and white stars indicate passion fruit, Arabidopsis and rice sequences, respectively; (**B**) Percentage of *LBD* gene family members in three species in each subgroup. The blue part represents the proportion of *LBD* gene in passion fruit, the orange part represents the proportion of *LBD* gene number in Arabidopsis and the green part represents the proportion of *LBD* gene in rice.

To explore the evolutionary relationship between passion fruit PeLBD proteins and LBD proteins of other species, we combined the amino acid sequences of 33 passion fruit *PeLBDs*, 43 Arabidopsis LBDs and 36 rice LBDs and constructed a maximum likelihood (ML) system developmental tree (Figure 2). According to the results of the phylogenetic tree, a total of 112 LBD proteins from three species can be phylogenetically divided into seven subgroups; Class Ia, Class Ib, Class Ic, Class Id, Class Ie and Class IIa and Class IIb (Table S3). There are 95 *LBD* gene members in Class I: passion fruit (27, 28.4%), Arabidopsis (37, 38.9%) and rice (31, 32.6%). There are 17 *LBD* gene members in Class II: passion fruit (6, 35.3%), Arabidopsis (6, 35.3%) and rice (5, 29.4%). Each species contains members of each subclass, suggesting that all seven subclasses are present in both monocotyledonous and dicotyledonous plants and they may all share a common ancestor. However, *PeLBDs* were absent in several minor subgroups, such as the *AtLBD9*-containing minor groups in Class Id, which only contained seven *OsLBDs* and four *AtLBDs*. The *AtLBD23*-containing minor subgroups in Class Ib also only contained two *AtLBDs* and one *OsLBD*. *AtLBD26*-containing minor subgroups in Class Ic contained six *AtLBDs* and one *OsLBD*. Notably, most sister pair clades consisted of *PeLBD*s and *AtLBD*s (eg., *PeLBD18*/*AtLBD25*, *PeLBD17*/*AtLBD27*, *PeLBD11*/*AtLBD30*, *PeLBD15*/*AtLBD37*, *PeLBD21*/*AtLBD4*, etc.), in addition, this result suggests that *PeLBD* and *AtLBD* genes are evolutionarily more closely related. 

### 2.3. Gene Structure and Conserved Motif Analysis of PeLBD Genes

**Figure 3:**
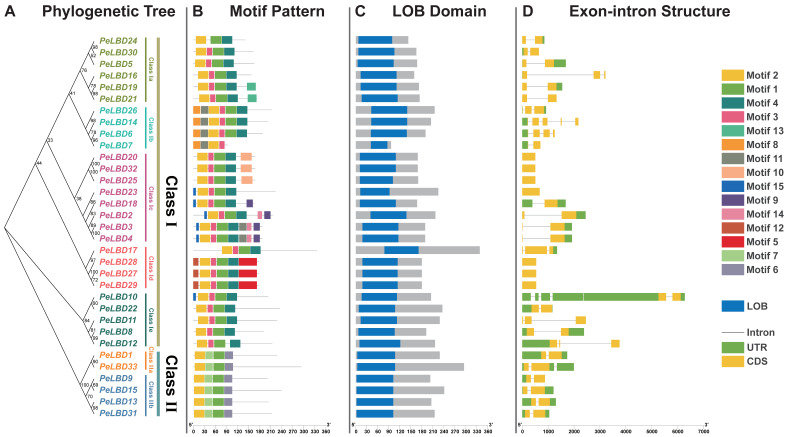
The phylogenetic relationship, conserved motifs and gene structure of PeLBD proteins. (**A**) The maximum likelihood (ML) phylogenetic tree of PeLBD proteins was constructed using full-length sequence with 1000 bootstrap replicates; (**B**) Distribution of conserved motifs in PeLBD proteins. A total of 15 motifs were predicted, and the scale bar represents 30 aa; (**C**) Distribution of LOB domain of *PeLBD* genes; (**D**) The gene structures of the *PeLBD* genes, include introns (black lines), exons (CDSs, yellow rectangles) and untranslated regions (UTRs, green rectangles). The scale bar indicates 1 kb.

Structural features of PeLBD proteins were investigated as a function of their phylogeny (Figure 3A). Consistent with the classification, Class I is divided into five subclasses, Ia, Ib, Ic, Id and Ie, with six, four, eight, four and five *LBD* gene family members, respectively. Class II is divided into two subclasses, IIa and IIb, with two and four *LBD* gene family members, respectively. Further, conserved motifs were predicted by MEME to understand the *PeLBDs* functional regions. A total of 15 conserved motifs in the PeLBD proteins were identified and named as Motifs 1-15 (Figure 3B). Motif 1 contains the CX2CX6CX3C zinc finger-like domain of the *LBD* gene family and was present in almost all of the PeLBD proteins except for *PeLBD7*, and Motif 2 was present in all of the PeLBD proteins, which constitute the most highly conserved part of the LOB domain (Figure 3C). Motif 3 and Motif 4 are only shown in the Class I *PeLBDs*. Motif 4 contains the LX6LX3LX6L leucine zipper-like domain which is absent from the Class II *PeLBDs* in agreement with the classification basis of LBDs, suggesting that the classification of *PeLBDs* in this work is quite reliable. Besides, Motifs 5–10 were specific to a particular subgroup; for example, Motif 6 widely appeared in the Class II genes but not Class I genes; Motifs 5 and 12 only appeared in Class Id; Motifs 9, 10 and 14 only existed in Class Ic genes; andMotifs 8 and 11 were present in the Class Ib genes. By analyzing the structure of *PeLBD* genes (Figure 3D), we revealed that the closely related gene members tended to show similar exon/intron structures. Apart from *PeLBD10* and *PeLBD33* (containing six and three introns, respectively), the other *LBD* genes did not contain more than two introns, and of them, nine genes did not contain any introns. *PeLBD* genes clustered in the same subclasses possess similar motif composition and gene structure, indicating that the phylogenetic relationship among *PeLBDs* is highly correlated with gene structures.

### 2.4. Analysis of Cis-Acting Elements in PeLBD Promoters

**Figure 4:**
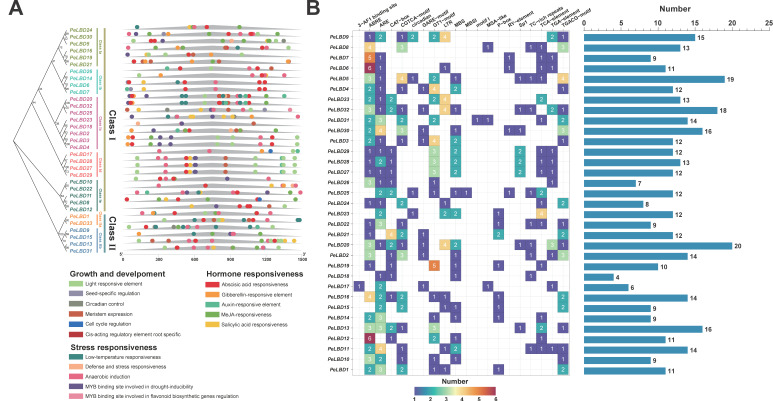
The *cis*-acting element on the putative promoter of the *PeLBD* genes. (**A**) Distribution of *cis*-acting elements identified in the 1500 bp upstream promoter region of *PeLBD* genes; (**B**) The number of *cis*-acting elements on putative promoters of *PeLBD* genes.

*Cis*-acting elements are non-coding DNA sequences in the gene promoter regions that regulate the transcription of their associated genes [29]. Here, the region 1500 bp upstream of the *PeLBD* gene transcription start site wase selected as the putative promoter region referred to by Huang et al. (2021) [30]. The putative promoter sequence of the *PeLBD* genes was then extracted and submitted to the PlantCARE database to search for *cis*-acting elements (Table S4), 20 representative *cis*-acting elements are shown in Figure 4A. In addition to the core *cis*-acting elements, many regulatory motifs were associated with light regulation (GT1-motif, 3-AF1 binding site, Sp1), low temperature (LTR), defense and stress responses (TC-rich repeats) and anaerobic induction (ARE). Besides, the MYB binding site involved in drought induction and gene regulation of flavonoid biosynthesis (MBS, MBSI); hormonal regulation such as salicylic acid (TCA-element); methyl jasmonate (e.g., CGTCA-motif, TGACG-motif); auxin (TGA-element); abscisic acid (ABRE); gibberellin (P-box, TATC-box, GARE-motif) and regulatory motifs related to tissue-specific expression (e.g., RY-element, CAT-box) or developmental processes/cell differentiation (e.g., MSA-like, circadian, Motif I) was also identified. Among those, the abscisic acid responsiveness elements were the most enriched *cis*-acting elements and widely distributed in Class I and Class II *PeLBD* promoter regions (including 28 *PeLBDs*), while auxin-responsive elements were mainly enriched in Class I *PeLBDs*. Some subclass gene members such as Class Ic possessed similar *cis*-acting elements in their promoter regions and all *PeLBDs* in this subgroup possessed MYB binding sites. In summary, these results may suggest that the functional expression of *LBD* genes in passion fruit is regulated by diverse *cis*-acting elements related to hormones, plant growth and development processes and stress response.

### 2.5. Chromosomal Location, Collinearity and Evolution Analysis of PeLBD Genes

**Figure 5:**
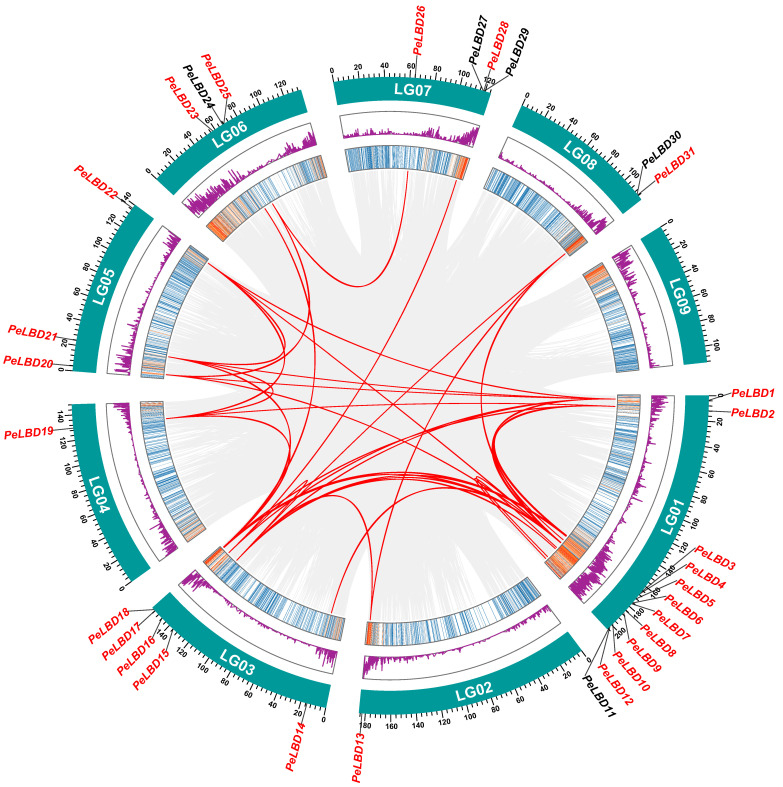
Distribution and collinearity of *PeLBD* gene family in passion fruit genome. *PeLBDs* marked in red have collinearity, while *PeLBDs* marked in black lack collinearity. The two rings in the middle represent the gene density of each chromosome. The gray background lines represent collinear backgrounds. The orange line represents the collinear relationship between *PeLBD* members.

According to available literature, the expansion of gene families is driven by different gene duplication patterns that are considered to be the driving force of species’ evolution [31]. Except for LG09, the *PeLBD* genes were unevenly distributed across nine linkage groups (LGs) of passion fruit (Figure 5). Among them, LG01 had the maximum number of *LBD* genes (12, 36.4%); followed by LG03 (5, 15.2%); LG07 (4, 12.1%); LG05 (3, 9.1%) and LG06 (3, 9.1%). It seemed that there was no positive correlation between LG length and the number of *LBD* genes, and there was no chromosomal bias observed in the distribution of the two classes of *PeLBDs*. By using MCScanX methods, there was only one tandem duplication event identified (*PeLBD11/12*), which was located on LG01. Besides, 26 *PeLBD* genes present on the duplicated segments of the passion fruit genome (Table S5), were matched as 43 segmental duplication gene pairs. These results indicated that some *PeLBDs* were possibly generated by gene duplication and the segmental duplication events played a major driving force for *PeLBDs* evolution. Furthermore, almost all the duplicated gene pairs belonged to Class I, which accounted for about 82% of the total number of *PeLBD* genes. To further explore the evolutionary process and selection pressure acting on the *PeLBD* genes, we calculated the Ka/Ks ratio of all the 44 duplicated *PeLBD* gene pairs. The Ka/Ks ratio of 44 *PeLBD* gene pairs was all less than 1 (Table S6), indicating that the *LBD* gene family of passion fruit might have experienced purifying selection during evolution. The purifying selection might have played a key role in maintaining the conserved structure of the *LBD* genes throughout evolution.

**Figure 6:**
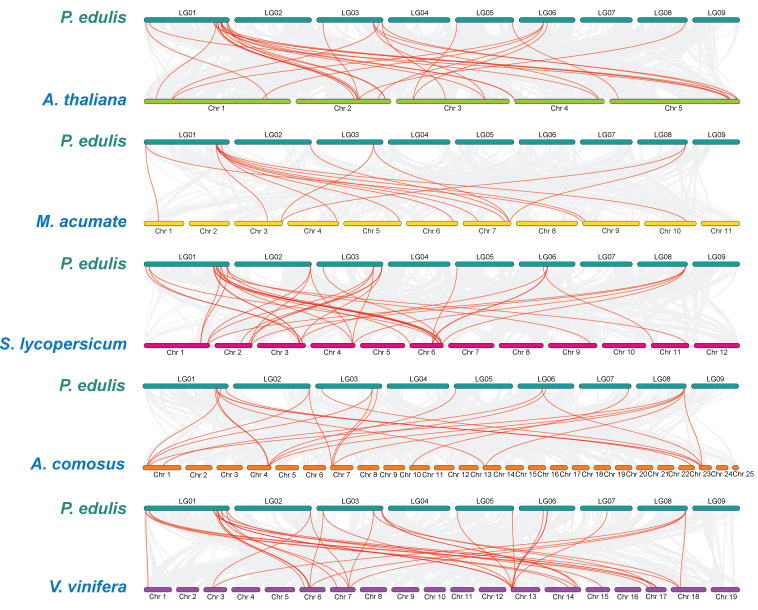
*LBD* genes synteny analysis of passion fruit and five representative plants. Grey lines in the background indicate collinear blocks in passion fruit and other plant genomes, while red lines highlight syntenic *LBD* gene pairs. Species names are prefixed with ‘*A. thaliana*’, ‘*M. acuminate*’, ‘*S.lycopersicum*’, ‘*A.comosus*’ and ‘*V.vinifera*’, denote *Arabidopsis thaliana*, *Musa acumate*, *Solanum lycopersicum*, *Ananas comosus* and *Vitis vinifera*, respectively.

Collinear analysis of different species is a way to study their evolution and affinities [32]. To further study the gene duplication timing of the *PeLBD* gene and infer its phylogenetic mechanism, we selected five representative species for comparative analysis of collinearity with passion fruit, including three dicots (Arabidopsis, tomato and grape) and two monocots (banana and pineapple) (Figure 6). A total of 23 *PeLBD* genes were collinear with grape genes, followed by tomato (22), Arabidopsis (20), pineapple (15) and banana (7), respectively. These results showed that the collinearity between the genomes of passion fruit and dicotyledons was greater than that between the genomes of passion fruit and monocotyledons. Among them, *PeLBD1*, *PeLBD6* and *PeLBD31*, for example, were associated with at least three isogenic pairs (especially in the *LBD* genes of passion fruit and grape), suggesting that these genes may have played important roles in the *LBD* gene family in the evolutionary process. 

### 2.6. Homology Modeling of PeLBD Tertiary Structures

**Figure 7:**
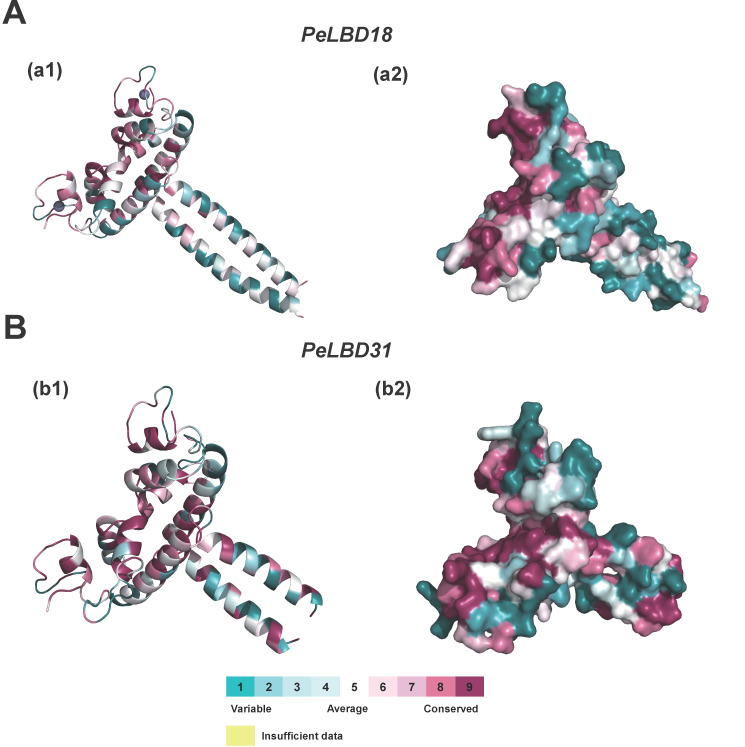
Predicted three-dimensional structures of the passion fruit LBD protein sequences. Display type: Cartoon (**a1**,**b1**); Surface (**a2**,**b2**). (**A**) *PeLBD18* represents the structure of the Class I subfamily; (**B**) *PeLBD31* represents the structure of the Class II subfamily.

Based on the SWISS-MODEL database, homologous modeling of PeLBD protein was carried out, and the member structures of the two subfamilies were predicted. The structure with the highest GMQE and QMean was selected as the best structure of PeLBD protein (Table S7). Therefore, *PeLBD18* from Class I (Figure 7A) and *PeLBD31* from Class II (Figure 7B) were selected as research targets. Each subfamily is composed of two chains, A and B, and presents a symmetrical “Y” structure. A similar “pocket” area is formed at the combination of the A and B chains, indicating that this area is highly conserved. At the same time, there are also some conserved structures in the amino acid terminal (NTR) and carboxyl terminal region (CTR) in each subfamily, which can be inferred that the genes and functions of the two subfamilies, Class I and Class II, are similar at the protein structure level [33].

### 2.7. Expression Patterns of PeLBDs in Different Tissues of Passion Fruit

**Figure 8:**
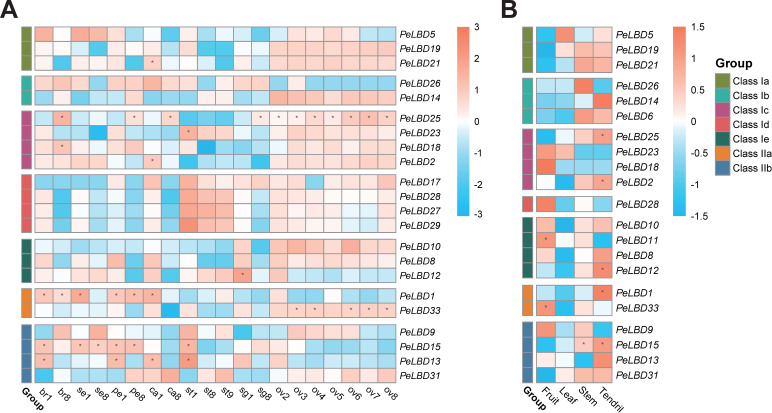
The expressional pattern of *LBD* genes in passion fruit. Heatmap of expression levels of 33 *PeLBD* genes in (**A**) floral tissues at different developmental stages. br, bract; se, sepal; pe, petal; ca, corona filament; st, stamen; sg, stigma; ov, ovule; numbers represent developmental stages, 1 and 2 was early stage, 8 was late stage (Table S9); (**B**) Other tissues including fruit, leaves, stems, and tendrils. The heatmap was created based on the log_2_(TPM + 0.01) value of *PeLBDs* and normalized by row. The TPM value higher than 32 was shown as abundant genes, and marked with “*”. Differences in gene expression changes are shown in color as the scale, coral for high expression and blue for low expression.

*LBD* genes are reported to be involved in many aspects of plant organ or tissue growth and development, including lateral roots, stems, leaves and flowers [9,10,11]. Here, the expression patterns of 33 *PeLBD* genes in floral tissues including ovule, bract, sepal, petal, corona filament, stamen and stigma at different developmental processes (Figure 8A), as well as other tissues such as the young leaf, stem, tendril and fruit (Figure 8B), were studied. Genes with low expression levels in all samples (all belonging to Class I) were filtered out. 

As shown in Figure 8A, 22 *PeLBDs* showed obvious different expression profiles across all floral tissues. *PeLBD13* was highly expressed in all the floral tissues at the early developmental stage. *PeLBD15* was highly expressed at the early developmental stage of bracts and stamens, as well as at all stages in sepals and petals. Similarly, *PeLBD1* was highly expressed at early developmental stage of sepals, corona filaments and at all stages of bracst and petals. In contrast, *PeLBD25* was highly expressed at the late developmental stages of bracts, petals, corona filaments, stigmas and during all stages of ovules. Except for these specific genes, some *PeLBDs* in the same subclass showed similar expression patterns in certain tissue development, such as all *PeLBDs* in Class Id showed higher expression levels in all stages of stamens with a decreasing trend. Meanwhile, *PeLBD23* in Class Ic also showed similar expression profiles and was highly expressed in stamens. Among all the remaining *PeLBDs* shown in the heatmap, most of them showed a higher expression pattern during ovule development, including *PeLBD19* and *PeLBD21* in Class Ia, *PeLBD14* with a descending trend in Class Ib, all the four remaining *PeLBDs* in Class Ic, *PeLBD10* and *PeLBD8* in Class Ie, *PeLBD33* in Class IIa and *PeLBD31* with an ascending trend in Class IIb. These results suggested that diverse *PeLBDs* might be involved in the floral development of passion fruit, and some of them might be redundant in function. 

In terms of other vegetative tissues, 21 *PeLBDs* were kept after filtering low expressed genes. A total of 14 out of the 21 *PeLBDs* were relatively more highly expressed in the tendrils, which are part of the *Passiflora* inflorescence [34]. There were also many *PeLBDs* showing higher expression levels in the stems, including *PeLBD19*, *PeLBD21*, *PeLBD26*, *PeLBD6* and *PeLBD15*. Only *PeLBD5* and *PeLBD23* were highly expressed in young leaves. As to fruit, *PeLBD23*, *PeLBD18*, *PeLBD28*, *PeLBD11*, *PeLBD33* and *PeLBD9* were highly expressed. 

Across all samples (Table S8), several *PeLBDs* showed a strong tissue specificity of expression. For example, *PeLBD6* in Class Ib was specifically highly expressed in the stems and tendrils. Class Id *LBD* genes, including *PeLBD17*, *PeLBD27* and *PeLBD29* were mainly highly expressed in stamens and ovules, while nearly not expressed at all in all the vegetative tissues. *PeLBD11* in Class Ie was specifically highly expressed in fruit. These results suggest that certain *PeLBD* genes might have a specific function in the development of corresponding tissues. 

**Figure 9:**
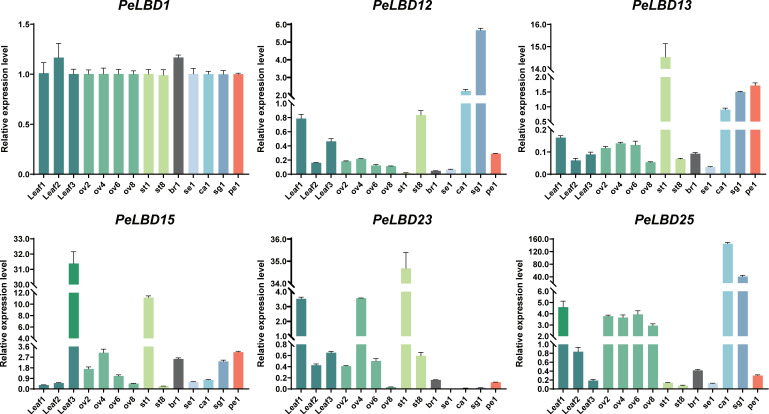
qRT-PCR analysis of six genes (*PeLBD1*, *PeLBD12*, *PeLBD13*, *PeLBD15*, *PeLBD23* and *PeLBD25*) in eight passion fruit tissues (leaf, ovule, bract, sepal, petal, corona filament, stamen and stigma). All experiments were performed independently at least three times. Error bars represent the standard deviation of three replicates.

To verify the reliability of the transcriptome data and explore the functions of *PeLBDs* during leaf development, the expression profiles of six representative *PeLBDs* in the same floral samples as transcriptome and leaves at three development stages (Leaf1-young leaf, Leaf2-light green leaf and Leaf3-dark green old leaf) were explored using qRT–PCR (Figure 9) (Table S10). Except for *PeLBD1* in Class IIa, which presented a broad expression pattern in both floral and vegetative tissues, the other five *PeLBDs* showed different expression patterns in different tissues. Consistent with the transcriptome data, *PeLBD12* was highly expressed at the early developmental stage of stigmas and corona filaments, as well as at the late developmental stage of stamens; the expression of *PeLBD13* was highest in the early stage of stamen development and relatively higher in the early stage of corona filament, stigma and petal development. The expression profile of *PeLBD15* in floral tissues was similar to *PeLBD13* but was expressed highest at the late developmental stage with an ascending trend during leaf development. *PeLBD23* was highly expressed in stamens with a descending trend during stamen development and also showed higher expression levels at the early developmental stage of leaf development. *PeLBD25* was consistently more highly expressed at the early developmental stages in diverse floral and vegetative tissues, but with a stable expression level during ovule development. Overall, the validation results using qRT-PCR supported the results of the transcriptome data analysis, and also suggested that *PeLBD15*, *PeLBD23* and *PeLBD25* might play important roles in leaf growth and development of passion fruit.

**Figure 10:**
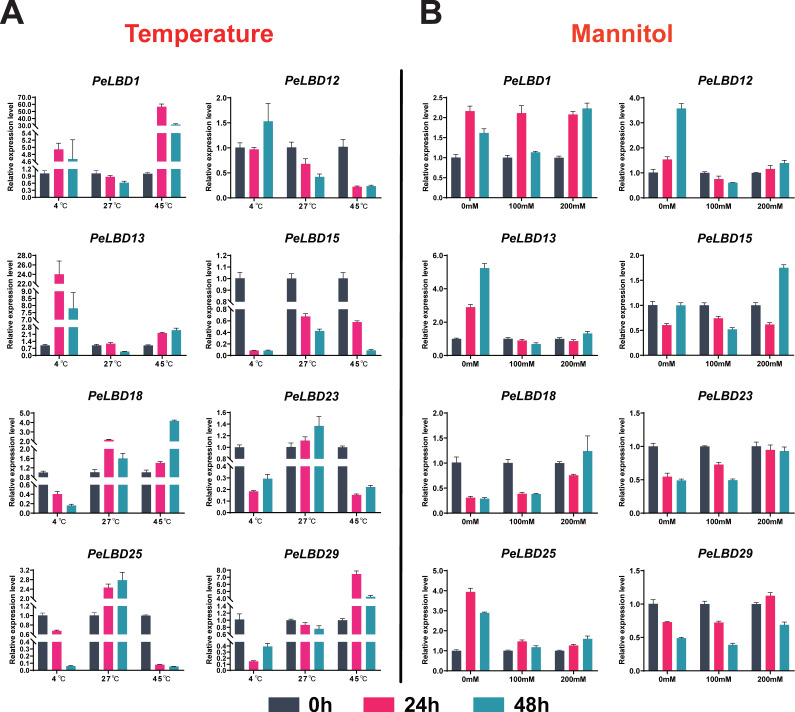
qRT-PCR analysis of eight genes (*PeLBD1*, *PeLBD12*, *PeLBD13*, *PeLBD15*, *PeLBD18*, *PeLBD23*, *PeLBD25* and *PeLBD29*) under (**A**) cold (4 °C), heat (45 °C) and (**B**) drought stress treatment. All experiments were performed independently at least three times. Error bars represent the standard deviation of three replicates.

To further inquire about the functions and utility of *PeLBD* genes correlated with their expression, eight representative genes were subjected to the qRT-PCR to scrutinize the expression profiles of *PeLBD* genes under cold, heat and drought stress treatments of passion fruit. Under cold stress (4 °C), *PeLBD1/12/13* were induced with higher expression levels compared with the control (27 °C) at 24 h and 48 h, while others were depressed. Under heat stress (45 °C), *PeLBD1* and *PeLBD29* were more highly expressed compared with the control at 24 h and 48 h, while *PeLBD12/23/25* were lowly expressed. Overall, the expression of all the detected eight *PeLBD* genes were influenced by temperature changes (Figure 10A). In order to observe the possible effect of *PeLBD* genes under drought stress, we conducted a drought stress trial using mannitol 100 mM and 200 mM concentration (Figure 10B). *PeLBD12/13/25* showed lower expression profiles under drought stress compared with the control, while *PeLBD15* was more highly expressed under mannitol 200 mM condition at 48h. *PeLBD1* and *PeLBD15* were nearly not affected by the drought stress at 24 h. On the basis of these findings, a potential role of *PeLBD* genes can be predicted against temperature and water scarcity conditions.

### 2.8. Identification and Annotation of PeLBD Target Genes

**Figure 11:**
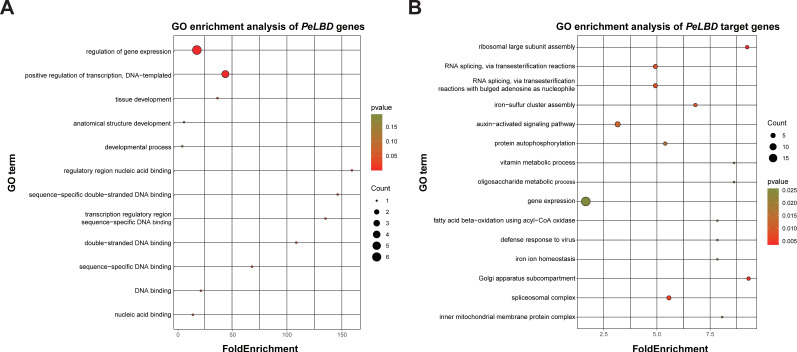
(**A**) Top 15 enriched GO terms for *PeLBD* genes; (**B**) Top 15 enriched GO terms for candidate *PeLBD* target genes. The horizontal axis represents the enrichment factor, and the size of the black circle indicates the number of genes annotated with a given GO term, and different colors represent *p*-values.

In order to explore the potential downstream target genes regulated by passion fruit *LBD* genes and determine their functions, 1500 bp upstream promoter sequence of passion fruit genes were submitted to the JASPAR database to detect the consensus LBD motifs. A total of 608 target genes were identified and further annotated (Table S11). A total of 483 genes obtained GO annotations and 147 genes were mapped to the KEGG database. To predict their biological functions, we first performed GO annotation and enrichment analysis on 33 PeLBD proteins; most of these genes were annotated with DNA binding or development-related terms, and three GO terms including regulation of gene expression, positive regulation of transcription, DNA-templated and tissue development were significantly enriched (Figure 11A). Meanwhile, the target genes of *PeLBDs* possessed significantly enriched biological processes such as ribosomal large subunit assembly, auxin-activated signaling pathway, gene expression, defense response to other organisms, regulation of growth, and diverse metabolic processes including vitamins, oligosaccharides, fatty acids and monocarboxylic acid (Figure 11B). These results suggested that *PeLBDs* could regulate multiple pathways of function by modulating their target genes.

## 3. Discussion

As a specific transcription factor (TFs) in plants, the *LBD* gene encodes a conserved LOB (lateral organ boundary) domain, which plays an important role in the growth and development of higher plants, including lateral organ development, abiotic stress reactions and metabolic processes, etc. At present, in view of the importance of the research value of the *LBD* gene family, it has been identified in different plants: Arabidopsis (43) [7], rice (36), maize (44) [35], grape (40) [26] and bayberry (33) [33]. Passion fruit is an exotic climbing vine with high economic, medicinal and ornamental value and also is a good model for floral development investigation. However, no research has been published on the *LBD* gene family of passion fruit. 

In this study, we identified 33 *LBD* genes from passion fruit, which were unevenly distributed on nine chromosomes, and classified them into Class I (27, 81.8%) and Class II (6, 18.2%) based on the presence of the conserved LX6LX3LX6L leucine zipper-like domain at the C-terminus. Similar to that in Arabidopsis and rice, the number of members of Class I was higher than that of Class II in the evolutionary process, and their phylogenetic relationship was basically the same as previous research results [8,36]. Homology modeling of two subfamily members of *PeLBD* proteins showed that each subfamily consists of two chains, A and B, in a symmetrical “Y” structure, and a similar “pocket” region is formed at the junction, indicating that the region is highly conserved. Based on the phylogenetic analysis of all 112 *LBD* genes from passion fruit (33), Arabidopsis (43) and rice (36), 33 *PeLBD* genes can be further divided into seven subclasses including Class I (a–e) and Class II (a–b). Compared with Arabidopsis (*A. thaliana*, 125 Mb, 25,498 genes) [37] and rice (*Oryza sativa* L. ssp. *Indica*, 430 Mb, 42,189 genes) [38], passion fruit possesses a larger genome with fewer protein-coding genes (1395.76 Mb, 23,171 genes) [39] showing fewer *LBD* gene family members. According to the ML tree of all LBDs in these species, we found that *PeLBDs* were absent in several minor subgroups, such as *AtLBD9*-containing minor groups in Class Id, which contains seven *OsLBD*s and four *AtLBDs*. The loss of *PeLBDs* in these minor subgroups might contribute to the overall low number of *LBD* gene members in passion fruit. 

The structural analysis could provide valuable information about the phylogenetic relationships of gene members within the same gene family and evolutionary duplication events. Motif and gene structure analysis showed that closely related gene members tended to show similar motif composition and exon/intron structure, as observed in other plants such as Arabidopsis and rice [8]. The conserved Motifs 1 and 2 observed at the N-terminus of the LOB domain region were present in almost all *PeLBDs*, which were critical for the functional specificities of these transcription factors family members [40]. The leucine zipper-like coiled-coil motif of Motif 4 was present only in all Class I *PeLBDs* but not Class II *PeLBDs*, indicating that the classification of Class I and Class II in the current study is reliable. Meanwhile, most of the other motifs, such as Motifs 5 and 6, were mainly observed in the highly variable C-terminal domain in PeLBD proteins, and family members within the same subclass generally possessed similar motif compositions. Gene exon/intron structure analysis showed that 94% (31 out of 33) of the *PeLBDs* contained no more than two introns, the same as the majority of *LBD* genes in other plants, suggesting a relatively conserved gene structure during evolution. Whereas gene family members in Class Ie showed obvious structural differences, and the number of introns contained in these *PeLBD* genes varied from one to six, suggesting that family members of Class Ie may have undergone splicing or insertion of gene segments during evolution [41]. Diverse motif composition and different gene structures among different subgroup members might contribute to the functional diversity of the *LBD* gene family [40]. 

As a key component of gene expression regulation, the binding between transcription factors (TFs) and *cis*-acting elements (or cis-regulatory DNA sequences) in the upstream promoter regions of genes plays an important role in the transduction of biological signals [42]. In this study, many regulatory motifs were identified in the putative promoter region of the *PeLBD* genes, and associated with hormonal regulation, growth and development and stress response. Among them, abscisic acid (ABA) responsiveness elements were the most enriched *cis*-acting elements and were widely distributed in most *PeLBDs* (29 out of 33). ABA is a well-known anti-stress plant hormone regulating diverse developmental processes and also plays a role in lateral root development regulation. In Arabidopsis, the expression of *AtLBD14* is downregulated by ABA during the entire steps of lateral root growth [43]. ABA can also interact with MYB transcription factors and play an important regulatory role in stress response [44]. The *LBD* gene family was also reported to be involved in abiotic stress response [45], and the MYB binding sites were also identified in the promoters of many of the *PeLBD* genes (19 out of 33). The abundant ABA responsiveness elements in most *PeLBDs* might be involved in the regulatory network of both plant development and stress response. Besides, auxin-responsive elements were mainly enriched in Class I *PeLBDs*. Functional analysis in Arabidopsis and other plants showed that Class I *LBD* genes are mainly involved in plant development, such as lateral root, leaf and flower development [11,20], while Class II *LBD* genes might be involved in metabolism-related processes [11,23]. The specific distribution of auxin-responsive elements in Class I *PeLBDs* is likely to play roles in establishing a molecular link between auxin signaling and plant development regulation. 

Collinearity analysis indicated that the expansion of the *PeLBD* gene family likely took place mainly by segmental duplication, as 43 segmental duplicated *PeLBD* gene pairs were identified while only one tandem duplicated *PeLBD* gene pair (*PeLBD11/12*) was found, similar to other species from different taxonomic groups [8,44,46,47]. Some duplicated genes, such as *PeLBD13* and *PeLBD15,* might show functional redundancy, as they were both clustered in Class IIb and also were similarly highly expressed at the early developmental stage of diverse floral tissues, including bracts, petals, stamens and ovules. Their further functional studies could help to unveil the evolutionary role of gene duplication and their contribution in plant processes. However, most of the duplicated gene pairs showed different expression patterns. For example, *PeLBD8* and *PeLBD12* in Class Ie were segmental duplicated gene pairs. *PeLBD8* was more highly expressed at the early development stage of bracts, petals, corona filaments, stigmas and all of the development stages of ovules, while *PeLBD12* was more highly expressed in stamens and lowly expressed in most developmental stages of ovules. Additionally, *PeLBD12* was tandem duplicated with *PeLBD11*, which is specifically more highly expressed in fruit. The expression divergence of these duplicated genes might suggest their functional diversification.

Floral tissue development could be used as a good model for lateral organ formation [48]. All the *PeLBDs* in Class Id showed higher expression levels during stamen development, which were clustered with *AtLBD27*. *AtLBD27* plays a key role in the asymmetric division of microspores and is crucial for pollen development in Arabidopsis [17]. The phylogeny and expression analysis of Class Id *PeLBD* genes suggest that these genes might also play important regulatory roles in pollen development in passion fruit. Expression analysis during floral tissues’ development processes also showed that all Class Ic and many other *PeLBDs* (including *PeLBD19*/*21* in Class Ia, *PeLBD14* in Class Ib, *PeLBD8/10* in Class Ie and *PeLBD33/31* in Class II) were relatively highly expressed during ovule development. Among these Class Ic genes, *PeLBD25* was also more highly expressed at the late developmental stage of bracts, petals and corona filaments; *PeLBD23* was also more highly expressed during stamen development with a decreasing trend; the other two were also relatively highly expressed in bracts, sepals and petals. In Arabidopsis, the Class Ic LOB gene AS2 (*AtLBD6*) associated with AS1 (an R2-R3 MYB-domain protein) and the JAGGED (JAG) genes function in the sepal and petal primordia to repress boundary-specifying genes for normal development of the organs. In stage 9 to 11 flowers, AS2 signals appeared in the adaxial side of petals as well as the ovules, suggesting the function of the AS1 and AS2 complex is likely regulated spatially and temporally during flower development [10]. These results suggested that *LBD* genes play important roles in ovule development in passion fruit and Class Ic *PeLBDs* might have a relatively conserved regulatory role during flower development. In addition to stamens and ovules, three genes, including *PeLBD12* in Class IIa and *PeLBD13/15* in Class IIb, also showed higher expression levels in other floral tissues. In Arabidopsis, Class II genes such as *AtLBD37*, *AtLBD38* and *AtLBD39* are negative regulators of anthocyanin biosynthesis and N availability signals [13]. Our expression analysis showed that Class II *LBD* genes in passion fruit might have evolved with different functions and many of them might be related to floral development. 

As to vegetative tissues, many *PeLBDs* genes were highly expressed in the tendril, which is reported as part of the *Passiflora* inflorescence [34]. Many *PeLBD* genes that were highly expressed in the floral tissues were also highly expressed in tendrils, such as *PeLBD19/21/14/25/2/10/8/12/1/15/13/31*, indicating tendril development might be closely associated with floral development. There were also obvious differences, such as *PeLBD6* was highly expressed in tendril and stem but expressed at really low levels during floral development. *PeLBD23/18/28/33* were highly expressed during ovule development but lowly expressed in tendril. The previous ultrastructural analysis shows that the tendril development is initially separated from the development of the flower. These different genes might be related to the inconsistent formation of tendrils and flower. The mechanisms by which *LBD* genes control leaf development have been well elucidated in Arabidopsis. Three *PeLBD* genes, including *PeLBD15* in Class IIa and *PeLBD23/25* in Class Ic, were more highly expressed during leaf development in passion fruit, only the two Class Ic genes showed a descending expression trend, namely highly expressed at the early development stage of leaves than the late stages. The homologous Class Ic *LBD* gene *AtLBD6/AS2* is specifically expressed in the adaxial side of leaves and regulates leaf formation [8]. That is, *PeLBD23/25* might be related to leaf early formation in passion fruit.

The development of passion fruit was highly influenced by ecological factors including light, temperature and water conditions. In plants, the LBD family transcription factors play important roles in development as well as in stress responses. According to the *cis*-element analysis, *cis*-elements such as MYB binding site involved in drought-inducibility and low-temperature responsiveness were distributed in the promoter region of many *PeLBD* genes such as *PeLBD1* and *PeLBD15* (Table S4). The expression profiles of representative *PeLBD* genes from Class I (*PeLBD12/18/23/25/29*) and Class II (*PeLBD1/13/15*) were explored by qRT-PCR as well. The expression of all *PeLBD* genes detected were altered with temperature changes, *PeLBD1* was induced by both cold and heat stress and showed higher expression levels, *PeLBD13* was induced by cold stress but depressed under heat stress, while the expression of *PeLBD18* and *PeLBD29* showed an ascending trend with the temperature increased. The diversity of the expression changes might indicate their different functions under climate changes. Compared with temperature treatment, fewer *PeLBD* genes showed obvious expression profile changes. *PeLBD12/13/25* were depressed under drought stress and showed lower expression levels, while *PeLBD1* and *PeLBD15* was nearly not affected by the drought stress at 24 h. Previous work showed that Class I and Class II *LBD* genes might function in different biological processes [11,20,23], but no clear differences between these two class *PeLBD* genes, neither in development regulation nor in stress responses, were found in this work.

To further explore the functions of *PeLBDs* and potential downstream target genes regulated by these *PeLBD* genes, GO annotation and enrichment analysis were performed. A total of 608 target genes were identified and mainly enriched in biological processes, including ribosomal large subunit assembly, auxin-activated signaling pathway, regulation of growth and diverse metabolic processes, including vitamins, oligosaccharides, fatty acids and monocarboxylic acid. These results provide more detailed information for us to reveal the functions of *PeLBDs*.

## 4. Materials and Methods

### 4.1. Identification and Sequence Analysis of LBD Proteins from Passion Fruit

Genomic data of passion fruit were downloaded from the National Genomics Data Center (NGDC) (https://ngdc.cncb.ac.cn/ (accessed on 1 February 2022)) and the login number is: GWHAZTM00000000. A hidden Markov model of the lateral organ boundaries domain (DUF260, PF03195) was obtained from the Pfam database (http://pfam.xfam.org/ (accessed on 1 February 2022)) and used as the seed model for an HMMER-3.3 search (http://hmmer.janelia.org/ (accessed on 1 February 2022)) of the local passion fruit protein database (*E* ≤ 10^− 20^) [49], and redundant genes were removed to produce a set of preliminary LBD candidate sequences. To verify that these candidates were LBDs, we used the NCBI Conserved Domain Database (https://www.ncbi.nlm.nih.gov/cdd/ (accessed on 1 February 2022)) (E-value < 1 × 10^−5^, other parameters set to defaults) and the SMART (http://smart.embl-heidelberg.de/ (accessed on 1 February 2022)) [50] to filter with the LOB domain sequence. The confirmed *LBD* genes were renamed according to their positions on the passion fruit chromosomes.

Subcellular localization predictions were generated with Cell-PLoc 2.0 (http://www.csbio.sjtu.edu.cn/bioinf/Cell-PLoc-2/ (accessed on 1 February 2022)) [51], and the ExPASy ProtParam tool (https://web.expasy.org/protparam/ (accessed on 1 February 2022)) [52] was used to predict protein physicochemical parameters such as molecular weight (MW), isoelectric point (PI) and grand average of hydropathicity (GRAVY).

### 4.2. Multiple Sequence Alignment and Phylogenetic Tree Construction

Whole genome information for Arabidopsis and rice was downloaded from the TAIR10 database (http://www.arabidopsis.org/index.jsp (accessed on 2 February 2022)) and the Rice Genome Annotation Project database (http://rice.plantbiology.msu.edu (accessed on 2 February 2022)), respectively. Banana, tomato, grapes and pineapple genomic data were downloaded from the Ensembl database (http://plants.ensembl.org/index.html (accessed on 2 February 2022)).

According to the Plant Transcription Factor Database (http://planttfdb.gao-lab.org/index.php (accessed on 2 February 2022)), there are 43 and 36 LBD proteins in Arabidopsis and rice, respectively [53]. The Arabidopsis and rice LBD sequences were combined with those from passion fruit, and the multiple protein sequence alignment was produced with MUSCLE [54]. The phylogenetic tree of related proteins was constructed by MEGA 11 software using the maximum likelihood method and the verification parameter bootstrap was set to 1000 [55]. The results of the evolutionary tree were visualized by Evolview (http://evolgenius.info/#/ (accessed on 3 February 2022)) for post-processing. The LBD protein sequences of conserved domains were compared and edited using Jalview software (V2.11.1.4) (http://www.jalview.org/ (accessed on 2 February 2022)) [56], and the Jalview output was submitted to JPred (http://www.compbio.dundee.ac.uk/jabaws (accessed on 2 February 2022)) for protein secondary structure prediction using default parameters [57]. The conserved motif Logos were generated with the WebLogo (http://weblogo.threeplusone.com (accessed on 2 February 2022)) [58].

### 4.3. Gene Structure, Conserved Motif and Cis-Regulatory Elements Analysis

The intron–exon distributions of the passion fruit *LBD* genes were obtained using GFF annotation files from the passion fruit genome. Protein sequence analysis of MEME online program (http://meme-suite.org/ (accessed on 3 February 2022)) [59] was used to identify the identified conservative motif in passion fruit LBD proteins. The optimized parameters were employed as follows: the number of repetitions, any; the maximum number of motifs, 15; and the optimum width of each motif, between 6 and 100 residues. TBtools (V1.0986) software was used to extract the 1500-bp promoter region upstream of each gene’s transcription start site of all *PeLBD* genes [60]. Then, PlantCare (http://bioinformatics.psb.ugent.be/webtools/plantcare/html/ (accessed on 4 February 2022)) was used to predict the *cis*-acting elements in the putative promoter region of *PeLBD* genes [61]. The results were visualized using TBtools [60] and the function pheatmap package in R (https://cran.r-project.org/web/packages/pheatmap/index.html (accessed on 4 February 2022)).

### 4.4. Chromosomal Distribution and Gene Duplication

All *PeLBD* genes were anchored to their corresponding chromosomes using Circos [62] against physical location information from the passion fruit genome database. To demonstrate the synteny of orthologous *LBD* genes obtained from passion fruit and other selected species, we analyzed gene duplication events by using the Multicollinearity Scanning Toolkit (MCScanX) [63], setting default parameters, and using Circos and Dual Synteny Plot visualization results in TBtools [60].

For Ka/Ks analysis, seventeen homologous gene pairs were identified by BLASTn using two criteria: (1) >75% sequence similarity and (2) an region able to be aligned >75% of the length of the longer sequence [64]. KaKs_Calculator2.0 was used to calculate the synonymous substitution rate (Ks), nonsynonymous substitution rate (Ka) and Ka/Ks ratio between homologous gene pairs [65]. Its parameters are set as: NG as the Method (−m) and Standard Code as the Genetic code table (−c). Evolutionary divergence times within the passion fruit *LBD* gene family were calculated using the passion fruit-specific divergence time formula T = Ks/2λ (where λ = 8.12 × 10^− 9^).

### 4.5. Three-Dimensional (3D) Structural Modeling of LBD Family Proteins

The PDB database (http://www.rcsb.org/ (accessed on 5 February 2022)) was used to retrieve protein models homologous to the LBD protein of passion fruit. Then the Swiss model was used with default parameters (https://www.swissmodel.expasy.org/ (accessed on 5 February 2022)) by homology modeling to predict the protein tertiary structure, and the ConSurf (https://consurf.tau.ac.il/ (accessed on 5 February 2022)) was used to analyze the structure of the conservative [66]. Finally, predicted model structures were visualized and manipulated with PyMOL [67].

### 4.6. Identification and Annotation of LBD Target Genes 

To obtain downstream target genes that may be regulated by the *LBD* gene, we utilized the 1500 bp upstream (putative promoter region) sequence of the previously obtained transcription start site, according to which the consensus motif of the LBD DNA binding site (MA1673.1) was derived from Obtained from the JASPA_CORE database (https://jaspar.genereg.net/ (accessed on 6 February 2022)) [68]. Subsequently, the Motif FIMO program in the MEME online program was used to detect the LBD-binding motifs shared by the promoter region of passion fruit, and the screening criteria of *p* < 1 × e^−6^ were set to determine the final candidate *LBD* target genes. Functional annotation of candidate *LBD* target genes was performed using GO and the Kyoto Encyclopedia of Genes and Genomes (KEGG) databases.

### 4.7. PeLBDs Transcriptome Analysis Based on RNA-Seq Data 

The purple passion fruit (*P. edulis* Sims) used in this study were planted in the orchard of the Institute of Horticulture, Guangxi Academy of Agricultural Sciences. Diverse floral and vegetative tissues at different developmental stages were sampled. All tissues were dissected by hand and frozen immediately in liquid nitrogen, and a dissecting microscope was used for the ovule tissues. For floral tissues’ sampling, buds with bracts were cut, the maximum length of the proximal-distal axis and the horizontal width were used as a reference for measurement. The full lengths from visible petals were measured. Different floral developmental stages were defined according to the ovule development stages shown in Table S9, and the corresponding bud sizes were collected. Diverse floral tissues at an early stage when the archesporial cell had formed and the late stages when ovules had fully differentiated were used for the RNA-seq. Meanwhile, other tissues including fruit, leaves, stems, and tendrils were also sampled at the same time at 110-day (fruit mature stage) post anthesis.

RNA extraction and Illumina sequencing were performed as previously described [69], with 1 μg RNA per sample and three independent biological replicates for each tissue. The cDNA libraries were constructed using the NEBNext Ultra™ RNA Library Prep Kit for Illumina (NEB, Beverly city, MA, USA), following standard protocols. The transcript abundance of *PeLBD* genes was calculated as TPM (Transcripts Per Kilobase Million). The heatmap was generated by pheatmap packages in R based the log_2_ (TPM + 1). GO annotation was extracted from the passion fruit genome functional annotation, and enrichment analysis was performed using R package ClusterProfiler [70].

### 4.8. Plant Material, Stress Treatment, RNA Extraction, and qRT–PCR Analysis 

The qRT–PCR analysis was used to verify the expression of *PeLBD* genes in diverse tissues using the same sample for RNA-seq. To explore the expression and potential regulatory roles during leaf development, leaf tissues at different development stages (Leaf1: young leaves, Leaf2: light green tender leaves and Leaf3: dark green old leaves) were collected from plants grown in the orchard of the Fujian Agriculture and Forestry University. 

Abiotic stress treatments (cold, heat and drought) were applied to fully grown (with well-developed roots and shoots) healthy and disease-free passion fruit plants and one control with three biological repeats. As to cold stress treatment, healthy plants were placed in a growth chamber with the temperature set at 4 °C; as to heat stress treatment, plants were kept in a growth chamber with the temperature set at 45 °C; as to drought stress, mannitol in concentrations of 100 mM and 200 mM were applied to healthy plants. The samples under stress treatments were collected at 24 h and 48 h time intervals, respectively, with normal plants that were not exposed to stress treatment used as the control. All the collected samples were immediately stored in liquid nitrogen prior to total RNA extraction.

The Trizol method (Invitrogen, Carlsbad, CA, USA) was used to extract total RNA, and the ThermoScript RT-PCR kit (Thermo Fisher Scientific, Carlsbad, CA, USA) was used to conduct the reverse-transcribed experiment. Real-time PCR was performed by using the SYBR Premix Ex Taq II system (TaKaRa Perfect Real Time) in the Bio-Rad Real-time PCR system (Foster City, CA, USA), and the primers used are listed in Table S10. The qRT-PCR program was: 95 °C for 30 s; 40 cycles of 95 °C for 5 s; 60 °C for 34 s; 95 °C for 15 s. The passion fruit EF1a was used as an internal control to normalize the mRNA levels [71]. For each analysis, three technical replicates from three biological replicates were performed, the fold change of genes was calculated using the 2^−ΔΔCT^ method.

## 5. Conclusions

In our study, a total of 33 *PeLBDs* were identified in the passion fruit genome, which were unevenly distributed on nine chromosomes, and we classified them into Class I (27) and Class II (6), according to phylogenetic and gene structure analysis. Closely related gene members tended to show similar motif composition and exon/intron structure. Homologous protein modeling results showed that the gene members of the two subfamilies were structurally and functionally similar. *Cis*-acting elements and target gene prediction analysis results suggested that *PeLBDs* might participate in various biological processes by regulating diverse target genes involved in growth and development, metabolism, hormone and stress response. Collinearity analysis indicated that the expansion of the *PeLBD* gene family probably took place mainly by segmental duplication, and some duplicated gene pairs, such as *PeLBD13/15,* might show functional redundancy, while most duplicated gene pairs, such as *PeLBD8/12,* showed different expression profiles indicating their functional diversification. Expression and phylogenetic analysis showed that Class Ic genes, including *PeLBD2/18/23/25* in passion fruit, might have a relatively conserved regulatory role in flower development and leaf formation, whereas Class II *PeLBDs* might have evolved with different functions and many of them might be related to floral development. Several *PeLBDs* showed a strong tissue specificity of expression, such as *PeLBD17/27/29* which were specifically expressed in floral tissues while *PeLBD11* was only highly expressed in fruit, indicating their specific function in the development of certain tissues. The qRT-PCR was conducted to verify the expression levels of six *PeLBDs* in diverse tissues and eight *PeLBDs* under abiotic stresses. These results provide valuable information for understanding the evolution of the *PeLBD* gene family and facilitate further research on the functional characterization of *PeLBDs* in floral and vegetative tissues’ development in future studies.

## Figures and Tables

**Table 1:** Detailed information on 33 *PeLBD* genes of passion fruit and their encoded proteins.

Gene Name	Gene ID	Chromosome	Size (aa)	MW (Da)	pI	A.I.	Stability	GRAVY	Predicted Location
*PeLBD1*	P_edulia010000163.g	LG01	228	24,788.28	6.7	92.37	U	−0.208	Nucleus
*PeLBD2*	P_edulia010000500.g	LG01	216	23,829.2	6.3	71.44	U	−0.26	Nucleus
*PeLBD3*	P_edulia010001973.g	LG01	188	20,672.23	5.64	68.56	U	−0.353	Nucleus
*PeLBD4*	P_edulia010002080.g	LG01	188	20,702.3	5.64	70.64	U	−0.324	Nucleus
*PeLBD5*	P_edulia010002578.g	LG01	166	18,452.21	5.36	85.3	U	−0.087	Nucleus
*PeLBD6*	P_edulia010002985.g	LG01	189	20,945.79	9.08	72.91	U	−0.441	Nucleus
*PeLBD7*	P_edulia010003009.g	LG01	95	10,772.56	9.64	57.58	U	−0.58	Nucleus
*PeLBD8*	P_edulia010003878.g	LG01	191	20,985.21	9.28	86.28	U	−0.121	Nucleus
*PeLBD9*	P_edulia010004119.g	LG01	202	21,696.82	9.37	83.56	U	−0.045	Nucleus
*PeLBD10*	P_edulia010005184.g	LG01	204	22,205.36	8.37	82.35	U	−0.107	Nucleus
*PeLBD11*	P_edulia010005391.g	LG01	228	24,231.57	7.63	77.94	U	−0.132	Nucleus
*PeLBD12*	P_edulia010005394.g	LG01	215	23,314.77	8.9	85.77	U	−0.17	Nucleus
*PeLBD13*	P_edulia020007264.g	LG02	205	22,251.98	6.95	62.78	U	−0.412	Nucleus
*PeLBD14*	P_edulia030008046.g	LG03	204	23,008.4	9.04	72.79	U	−0.45	Nucleus
*PeLBD15*	P_edulia030008474.g	LG03	240	26,282.99	8.05	76.75	U	−0.275	Nucleus
*PeLBD16*	P_edulia030008742.g	LG03	158	17,413.83	7.64	72.91	U	−0.333	Nucleus
*PeLBD17*	P_edulia030009145.g	LG03	337	38,858.07	8.74	71.78	U	−0.612	Nucleus
*PeLBD18*	P_edulia030009282.g	LG03	166	18,330.5	5.93	62.95	U	−0.499	Nucleus
*PeLBD19*	P_edulia040010647.g	LG04	171	18,559.21	8.24	71.35	U	−0.258	Nucleus
*PeLBD20*	P_edulia050011298.g	LG05	168	18,645.42	7.52	74.94	U	−0.181	Nucleus
*PeLBD21*	P_edulia050011880.g	LG05	173	18,601.25	8.59	73.93	U	−0.205	Nucleus
*PeLBD22*	P_edulia050012869.g	LG05	235	25,742.65	4.5	75.23	U	−0.161	Nucleus
*PeLBD23*	P_edulia060015822.g	LG06	224	24,306.31	8.25	71.61	U	−0.401	Nucleus
*PeLBD24*	P_edulia060015864.g	LG06	142	15,627	7.61	82.46	U	−0.062	Nucleus
*PeLBD25*	P_edulia060015871.g	LG06	169	18,867.63	7.52	72.78	U	−0.227	Nucleus
*PeLBD26*	P_edulia070017232.g	LG07	214	23,195.4	8.83	72.57	U	−0.3	Nucleus
*PeLBD27*	P_edulia070018122.g	LG07	179	20,573.48	6.22	88.27	U	−0.419	Nucleus
*PeLBD28*	P_edulia070018138.g	LG07	179	20,557.48	6.22	88.27	U	−0.396	Nucleus
*PeLBD29*	P_edulia070018148.g	LG07	179	20,573.48	6.22	88.27	U	−0.419	Nucleus
*PeLBD30*	P_edulia080020093.g	LG08	164	18,188.81	6.8	87.44	U	−0.17	Nucleus
*PeLBD31*	P_edulia080020165.g	LG08	214	23,141.16	8.62	66.07	U	−0.379	Nucleus
*PeLBD32*	P_eduliaContig20022702.g	Contig2	168	18,617.36	6.71	74.94	U	−0.175	Nucleus
*PeLBD33*	P_eduliaContig60022431.g	Contig6	294	31,614.88	8.75	71.67	U	−0.402	Nucleus

MW, molecular weight; pI, isoelectric point; A.I, aliphatic index; GRAVY, grand average of hydropathicity score.

## Data Availability

The data presented in this study are available in the article, Supplementary Materials and online repositories. RNA-seq data used in this work were deposited in China National GenBank (CNGB) under accession number CNP0002747.

## Supplementary Materials

The following supporting information can be downloaded at: https://www.mdpi.com/article/10.3390/ijms23094700/s1.
